# Malignant catatonia in an adolescent with pogo transposable element derived with zinc finger domain (POGZ) gene mutation: case report

**DOI:** 10.1192/bjo.2025.10807

**Published:** 2025-08-01

**Authors:** Liron Leibovitch, Alon Gorenshtein, Erez Bibi, Ayala Uri

**Affiliations:** Department of Medicine, Azrieli Faculty of Medicine, Bar-Ilan University, Safed, Israel; Department of Child and Adolescent Psychiatry, Ziv Medical Centre, Safed, Israel

**Keywords:** Malignant catatonia, POGZ gene mutation, adolescent, electroconvulsive therapy, case report

## Abstract

**Background:**

Malignant catatonia represents a severe and life-threatening neuropsychiatric syndrome that demands prompt recognition and intervention. This condition poses particular diagnostic and management challenges in adolescents, especially when genetic predispositions and neurodevelopmental vulnerabilities complicate the clinical picture.

**Aims:**

This report examines a complex case of malignant catatonia in a 17-year-old female with developmental delay but no prior psychiatric diagnoses, who developed severe cognitive and behavioural deterioration. We explore the diagnostic complexities, therapeutic challenges and potential genetic contributions to her presentation.

**Method:**

We present a comprehensive case analysis documenting clinical progression, treatment responses and genetic findings through whole-exome sequencing. The patient’s journey spans from initial presentation to long-term follow-up, with systematic assessment using standardised catatonia rating scales.

**Results:**

The patient’s condition manifested as severe psychomotor impairment, mutism and autonomic instability, showing minimal response to initial treatment. Electroconvulsive therapy yielded significant but temporary amelioration of symptoms. Genetic analysis revealed a heterozygous mutation in the pogo transposable element derived with zinc finger domain (POGZ) gene – a gene implicated in neurodevelopmental disorders – suggesting this variant contributed to her neurobiological vulnerability. Concurrent features of functional neurological disorder further compounded the diagnostic complexity, illustrating the intricate interplay between genetic susceptibility and clinical presentation.

**Conclusions:**

This case illuminates the challenges clinicians face when diagnosing and treating complex neuropsychiatric presentations in adolescents, particularly when genetic predispositions intersect with functional neurological symptoms. The findings emphasise how comprehensive, multidisciplinary approaches remain essential for optimal patient care. Moreover, this case highlights the selective utility of genetic investigation in elucidating potential underpinnings of complex, treatment-resistant malignant catatonia, whilst demonstrating that genetic variants may confer vulnerability rather than direct causation.

Catatonia, a complex neuropsychiatric syndrome characterised by motor, behavioural and emotional symptoms,^1^ affects approximately 10% of psychiatric in-patients, with a higher prevalence in mood disorders and autism spectrum disorders.^2,3^ Malignant catatonia, its most severe variant, poses significant diagnostic and management challenges, especially in adolescents.^4^

Diagnosing catatonia within the framework of neurodevelopmental disabilities (NDDs) presents considerable challenges. Notably, the hallmark features of catatonia – such as mutism, stereotypic movements and resistance to instructions – often overlap with the primary symptoms of NDDs. This is particularly evident in autism spectrum disorder (ASD), where catatonia affects a significant minority.^5–7^ The pogo transposable element derived with zinc finger domain (POGZ) gene, vital for chromatin remodelling and neurodevelopment,^8^ has mutations linked to White–Sutton syndrome, ASD, intellectual disability and other developmental phenotypes.^9–11^ However, defining direct causal relationships between genetic variants and acute neuropsychiatric syndromes is a complex endeavour.^12^

Diagnosing adolescent catatonia is complicated by symptom overlap with various neurological and psychiatric disorders, often causing misdiagnosis and treatment delays.^13–16^ This diagnostic challenge is heightened in patients with genetic disorders. While POGZ mutations are associated with NDDs,^17–19^ they are believed to contribute to a neurobiological vulnerability that may increase the risk for severe neuropsychiatric syndromes rather than be a direct cause.^20–22^ Thus, POGZ variants require careful clinical interpretation. Prompt identification and intervention are crucial for catatonia, especially its malignant variant. While benzodiazepines are first-line treatments, electroconvulsive therapy (ECT) shows high efficacy, particularly for treatment-resistant cases. Despite its established efficacy, paediatric ECT use remains low – for instance, only 0.03% of US youth in-patient admissions in 2019 involved ECT^23^ – and is constrained by legal, practical and regulatory hurdles, including a scarcity of specialised facilities and treatment delays.^24^ The persistent and relapsing nature of catatonia requires long-term management approaches.

This report details a complex case of malignant catatonia in a 17-year-old female with a background of neurodevelopmental delay and a newly identified, likely pathogenic de novo POGZ gene mutation. We explore the diagnostic and therapeutic complexities and underscore the necessity of a multidisciplinary team. Furthermore, we discuss the POGZ variant as a potential contributor to an underlying neurobiological vulnerability that may influence severe, treatment-resistant presentations of catatonia. This case aims to contribute to the understanding of the expanding clinical spectrum associated with POGZ mutations and to illustrate the potential value of genetic investigation in select, complex neuropsychiatric cases, rather than advocating for routine screening.

## Case presentation

**Fig. 1:**
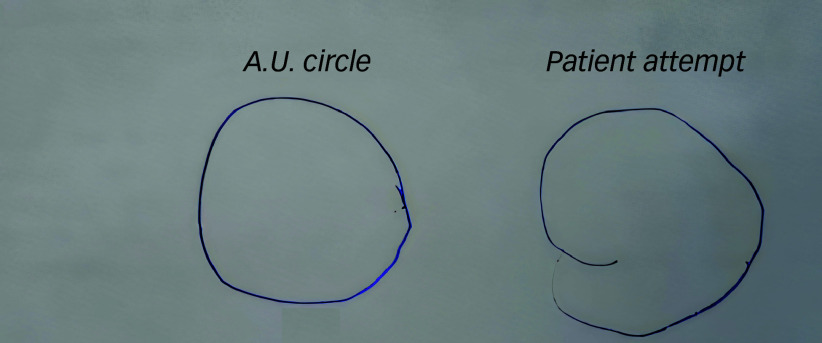
Comparison of circle drawings: physician’s demonstration (left) versus patient’s attempt (right).

In July 2019, a 17-year-old female, with early developmental delays but no prior psychiatric diagnoses, presented with her mother at our child and adolescent psychiatric clinic. Her mother reported a four-month history of significant cognitive and behavioural regression, characterised by disorientation, severely limited verbal communication, confusion (e.g. difficulty recognising family members), emotional lability and insomnia. Her cognitive difficulties were highlighted by her inability to draw a simple circle (Fig. 1).

Past medical history included being overweight and early developmental delays requiring physiotherapy and speech therapy. Neuropsychological assessment at age 12 revealed borderline intellectual functioning with graphomotor difficulties; ASD was excluded. Psychosocial factors included a challenging home environment (intellectually disabled siblings, father with chronic illness) and declining academic and social engagement in middle school, exacerbated by an undesired vocational high school placement at age fourteen, prompting school refusal. At age 16, an unverified report of being pursued by a partially clothed man coincided with the onset of anxiety regarding males.

By early May 2019, she developed mutism, social withdrawal and repetitive verbalisations (e.g. ‘no, no’ or ‘go, go’). Increasing confusion led to admission to a paediatric ward for several days in late May 2019 for diagnostic evaluation. A neurological examination indicated psychomotor impairment, temporal disorientation (place orientation intact) and blunted affect. Comprehensive initial investigations, including brain magnetic resonance imaging (MRI), electroencephalograms (EEGs), cerebrospinal fluid (CSF) analysis (negative for autoimmune encephalitis antibodies), 48-hour video EEG and extensive blood tests (ruling out metabolic, endocrine and autoimmune conditions like Hashimoto’s thyroiditis and porphyria), were unremarkable, revealing only low iron and folate. No specific pharmacological treatment was administered during this brief diagnostic admission.

In July 2019, out-patient follow-up revealed severe disorientation across all domains. Motor examination showed generalised rigidity requiring assistance for ambulation and sustained uncomfortable postures. Communication was minimal with profound comprehension deficits. Assessment revealed prolonged fixed staring, pronounced negativism and occasional contextually inappropriate echolalia. External stimuli elicited minimal reactions except in response to intense auditory or tactile prompts. No hallucinations were observed. Her Bush–Francis Catatonia Rating Scale (BFCRS) score reached 20, indicating severe catatonia. The clinical team assessed this catatonic presentation, examining underlying causes, including bipolar disorder, schizophrenia, dementia and primary neurological conditions, recognising that contextual symptom variability suggested potential functional overlay. An oral lorazepam dose of 1 mg was administered, and an urgent neuropsychiatric consultation was arranged.

The patient was admitted to the paediatric ward. Following the standard of care for catatonia, intravenous (i.v.) lorazepam was initiated and titrated upward based on clinical response, up to 4 mg three times daily. Despite the therapeutic intent, dose escalation resulted in paradoxical agitation. The patient’s presentation – marked by severe psychomotor impairment, mutism, prominent cognitive decline (including disorientation and confusion), bizarre behaviours and agitation – created significant diagnostic uncertainty due to substantial feature overlap between severe catatonia and an acute psychotic process. Consequently, an underlying psychotic disorder was considered a plausible differential diagnosis. Since the family refused ECT at this juncture, a trial of olanzapine was initiated, starting low and titrated upwards. Minor, transient activities of daily living (ADL) improvements at 10–15 mg daily proved inadequate, prompting escalation to 30 mg daily for 2–3 weeks – a regimen chosen despite its deviation from standard catatonia guidelines, reflecting the diagnostic complexity and urgent treatment constraints. Significant deterioration followed, including global aphasia and severe cognitive impairment. A mini-mental state examination (MMSE) of 4 out of 30, though limited by catatonia, documented this profound cognitive impairment. Concurrently, catatonic symptoms intensified, culminating in mutism and marked psychomotor impairment. Due to clinical worsening and heightened anxiety, olanzapine was discontinued, and fluoxetine (30 mg daily) was initiated.

By March 2020, her condition had deteriorated significantly; she could no longer walk independently and needed help with daily activities. In May 2020, she developed left-hand spasticity, leading to her readmission, with a BFCRS score of 24. Despite receiving i.v. lorazepam at 4 mg three times daily, she transitioned to malignant catatonia, characterised by both autonomic and psychomotor decline. Autonomic instability included fever (38.5°C), intermittent tachycardia, apnoeic episodes and fluctuating blood pressure. Worsening psychomotor symptoms included extreme negativism, active resistance, total mutism with echolalia, and rigid posturing, particularly of the left hand. Severe immobility necessitated percutaneous endoscopic gastrostomy (PEG) tube placement for nutritional support. She subsequently experienced complete withdrawal and became dependent on all ADLs. In response to her progression to malignant catatonia, bilateral ECT commenced, utilising brief pulse stimulation three times a week while under anaesthesia at a 1.5-fold threshold. Comprehensive investigations into potential underlying causes, including paraneoplastic aetiologies, were conducted using ultrasound, repeated EEGs and MRIs, all of which returned normal results.

After 11 ECT sessions, significant improvements emerged with her BFCRS score decreasing to 10. Motor functions enhanced markedly, allowing independent ambulation and resolution of left-hand posturing. Communication skills progressed to the formation of relevant sentences, despite persistent stuttering. Cognitive improvements included humour comprehension, partial orientation restoration and enhanced emotional regulation, evidenced by spontaneous smiling and increased social engagement. As treatment continued, clinical improvement accelerated with her BFCRS reaching a nadir of 2. Ongoing maintenance ECT was continued through July 2020, totalling 31 out-patient sessions, with BFCRS fluctuating between 5 and 7. In July 2020, she momentarily broke her mutism, discussing past bullying incidents by her classmates. Nevertheless, ECT was stopped due to rising seizure thresholds and a decline in efficacy, despite adjustments to the stimulation. Following this, clinical deterioration occurred rapidly, with the BFCRS rising to 18 within a few weeks. Her regression was evident through renewed feeding difficulties and severe psychomotor symptoms, including intense rigidity and posturing, which required complete physical assistance for all movements. Her severe clinical presentation, neurodevelopmental history and treatment-resistant catatonia prompted further aetiological investigation.

Following rapid clinical deterioration after ECT cessation in July 2020, the clinical team initiated whole exome sequencing (WES) to investigate potential genetic underpinnings of her treatment-resistant catatonia. Results identified a heterozygous POGZ mutation (c.2T>C;p. Met1?) at a conserved start codon, classified as likely pathogenic by multiple prediction algorithms. This mutation demonstrated variable expressivity, being present in both her father (who exhibited psychiatric symptoms and polyneuropathy) and her unaffected sister. No additional pathogenic variants were detected.

In August 2020, with severe symptoms persisting (BFCRS 18), she was transferred to another adolescent unit for diagnostic reassessment. Assessment revealed profound mutism with notable contextual variability. Identified stressors included an unverified incident with a partially clothed man and school bullying. Examination showed modifiable symptoms; rigid postures altered with suggestion and distraction, with functional improvement in familiar settings but deterioration in novel environments.

This pattern, coupled with premorbid vulnerabilities – academic difficulties, social isolation, family complexities, including intellectually disabled siblings and paternal chronic illness – suggests a functional overlay. While symptom modifiability and psychological triggers implied functional aspects, autonomic instability and ECT response strongly indicated primary catatonia. Overall, the clinical presentation suggests that these processes may not be distinct; psychological factors appear to influence the development and persistence of predominantly catatonic symptoms.

In October 2020, at the age of 18, the patient was admitted to internal medicine, experiencing profound unresponsiveness, mutism and an inability to eat or drink, necessitating the placement of a feeding tube. Due to paradoxical agitation in response to higher doses of lorazepam and concerns about respiratory depression, a conservative treatment approach was implemented, administering i.v. lorazepam up to 12 mg daily along with low-dose chlorpromazine (75 mg) for sedation. However, neither treatment improved her condition, leading to their discontinuation. Symptom fluctuations and transient post-medication improvements prompted reassessment for seronegative autoimmune encephalitis. A third CSF analysis revealed normal parameters, with negative autoimmune panels, consistent with the results of the two previous examinations. Based on the characteristic pattern of symptom variability, clinicians initiated empiric immunosuppression with i.v. methylprednisolone (1000 mg daily for 5 days) followed by oral prednisone (40 mg daily). This intervention improved oral intake, enabling discharge without the need for a feeding tube.

However, within weeks, her feeding capacity deteriorated significantly, necessitating PEG placement in December 2020 before her transfer to rehabilitation in early 2021. Her treatment regimen included oxazepam, amantadine and comprehensive rehabilitation therapy. Clinical progress occurred gradually; by August 2022, she demonstrated assisted ambulation and partial oral intake with minimal social engagement. A follow-up in November 2023 revealed substantial improvement, with independent mobility and appropriate self-care, although maternal assistance remained necessary for effective communication. Cognitive assessment revealed partial orientation (awareness of the day but not the month or year) and persistent memory deficits. Her BFCRS score of 10 indicated residual catatonic symptoms. Communication followed characteristic patterns: predominantly single words or brief phrases directed exclusively to family members, selective responsiveness to maternal instructions with inconsistent responses to others, and reliance on gestures for expressing basic needs. Nonverbal communication remained confined mainly to familial interactions. The patient’s complete clinical trajectory is summarised in Table 1.

**Table 1:** Patient clinical course and treatment progression

Date	Clinical presentation	Interventions	Outcome
Jul 2018	Onset of symptoms: disorientation, limited verbal communication	None	Spontaneous partial recovery
May 2019	Social withdrawal, minimal communication, perseverative speech patterns; hospitalisations: confusion, difficulty recognising family members, emotional distress	Neurological evaluation: brain MRI, EEG, and lumbar puncture with autoimmune panel	Inconclusive results: no abnormalities found
Jul–Sep 2019	Severe agitation, disorientation, minimal speech, severe comprehension impairment; hospitalisation: worsening symptoms; global aphasia, MMSE score of 4 out of 30	Lorazepam initiated and subsequently increased to i.v. (up to 4 mg TID); olanzapine introduced (up to 30 mg daily); lorazepam later discontinued	Minimal improvement followed by regression; transient improvement in ADLs following olanzapine introduction, followed by regression; global aphasia and severe cognitive decline developed
Mar–May 2020	Severe deterioration: loss of independent ambulation, spastic posture in left hand; malignant catatonia diagnosed: severe autonomic instability (fever, tachycardia, apnoeic episodes); BFCRS score of 24	Lorazepam (continued); ECT initiated due to progression to malignant catatonia and lack of response to lorazepam; extensive investigations^a^	Further decline; significant improvement after 11 ECT sessions: began walking, smiling and communicating in sentences (speech remained stuttered and slow); BFCRS decreased to 10; normal investigation results
Aug 2020	Post-ECT improvement; BFCRS score fluctuated between 5 and 7 (‘waxing and waning’ course)	ECT continued (total 31 sessions)	Temporary relief, walking and communication improved; BFCRS reached nadir of 2
Oct–Nov 2020	Severe decline: profound unresponsiveness, mutism, inability to eat or drink, tremors; transfer to adolescent psychiatry ward: profound communication barriers; BFCRS score of 18	Hospitalisation: feeding tube inserted; i.v. lorazepam (up to 12 mg daily), chlorpromazine 75 mg; i.v. methylprednisolone, followed by oral prednisone; WES	Minimal improvement; transient improvement in ability to eat following medication cessation; POGZ gene mutation (c.2T>C;p. Met1?) identified
Jan 2021	Ongoing catatonia; admitted to a rehabilitation centre	Oxazepam, amantadine, PEG nutrition, multidisciplinary rehabilitation initiated	Gradual improvement
Nov 2023	Clinical progress: walking unaided (accompanied by mother), partial verbal communication, partial orientation, impaired memory, BFCRS score of 10	Continued rehabilitation and follow-up	Significant improvement but ongoing cognitive limitations

ADLs, activities of daily living; BFCRS, Bush–Francis Catatonia Rating Scale; ECT, electroconvulsive therapy; EEG, electroencephalogram; i.v., intravenous; MMSE, mini-mental state examination; MRI, magnetic resonance imaging; PEG, percutaneous endoscopic gastrostomy; POGZ, pogo transposable element derived with zinc finger domain; TID, three times a day; WES, whole exome sequencing.

a .Investigations included ultrasound, EEG and MRI.

## Discussion

This case illustrates the diagnostic complexity of malignant catatonia against a background of neurodevelopmental vulnerability. The patient’s precipitous deterioration necessitated meticulous differential diagnosis beyond cardinal catatonic features. Environmental modifiability of symptoms – whereby emotional context influenced manifestation intensity and postural abnormalities demonstrated suggestibility – indicated functional neurological elements superimposed upon the underlying catatonic syndrome. Neurodegenerative aetiologies, though considered, were rendered less probable by the patient’s adolescent age, unremarkable neurodiagnostic results and characteristic symptom evolution.

The diagnostic formulation required careful distinction between catatonic and functional components. However, the notable autonomic instability, marked by fever, tachycardia and apnoeic episodes that necessitated PEG placement, coupled with a strong response to ECT, ultimately confirmed catatonia as the primary pathology. Recent literature elucidates how neurodevelopmental vulnerabilities may predispose an individual to catatonic and functional neurological presentations, potentially through shared neurobiological mechanisms involving striatal-thalamic-cortical circuits.^3,7^ While recognising this complex interplay, our management prioritised evidence-based interventions for life-threatening malignant catatonia while concurrently integrating psychological formulations to inform subsequent rehabilitation strategies, thereby addressing both the immediate autonomic instability and underlying psychological factors.

The course of treatment highlights the significant challenges in managing medications for evolving catatonia, especially concerning antipsychotic drugs. The initial uncertainty in diagnosis, combined with paradoxical agitation to lorazepam and the family’s initial reluctance towards ECT, prompted a trial of high-dose olanzapine. Unfortunately, this led to a clinical decline, illustrating iatrogenic exacerbation of catatonia due to antipsychotic use.^25^ This recognised complication was resolved after discontinuing the medication and starting evidence-based treatments, particularly benzodiazepines^26^ and ECT.^27^ This clinical pattern reinforces existing guidelines that emphasise established treatments for catatonia and recommend avoiding antipsychotics in the acute phase.^25^

Even with normal results from neuroimaging, EEG and lumbar punctures – showing no apparent cause – the rapid onset of symptoms, their severity and signs of autonomic instability strongly suggest malignant catatonia. The patient’s profound psychomotor impairment, disorientation and autonomic dysfunction reflect disruptions in cortico-striatal-thalamic circuitry implicated in catatonia pathophysiology.^28^ The multidisciplinary approach provided crucial diagnostic refinement by enabling concurrent neurological and psychiatric assessment, facilitating distinction between primary neurological disorders and the characteristic catatonic syndrome with functional overlay. This integrated evaluation, although not resolving all diagnostic uncertainties, enabled the timely implementation of life-saving interventions for malignant catatonia, demonstrating the value of collaborative care in complex neuropsychiatric presentations.^13^

The identification of a heterozygous POGZ mutation (c.2T>C; p. Met1?) affecting a highly conserved start codon^29^ provides valuable context, although it does not directly explain the causal mechanism underlying this complex presentation. The variable expressivity of this mutation, present in both the symptomatic father and asymptomatic sister, illustrates the nuanced relationship between genotype and psychiatric phenotype commonly observed in neurodevelopmental disorders.^18^ POGZ mutations disrupt chromatin remodelling during critical neurodevelopmental stages.^8,28^ potentially fostering a neurobiological vulnerability to severe psychiatric conditions, such as this patient’s catatonia, rather than directly triggering acute syndromes like catatonia itself. This concept aligns with contemporary theories on gene–environment interaction in psychiatry^20,21^ and is highlighted by epidemiological research showing that approximately one-third of paediatric catatonia cases arise in individuals with existing developmental disorders.^28^ Our patient’s presentation differs notably from the typical White–Sutton syndrome phenotype previously associated with catatonia,^10^ suggesting an alternative pathophysiological mechanism. Unlike SHANK3 mutations that demonstrate direct and consistent associations with catatonia through specific synaptic mechanisms,^30^ POGZ variants appear to confer broader neuropsychiatric susceptibility through altered neurodevelopmental architecture.^19^ This case contributes to the expanding POGZ-related clinical spectrum^9,17^ and illustrates the potential utility of considering comprehensive genetic evaluation in select cases of severe, treatment-resistant catatonia, particularly when occurring against a background of neurodevelopmental vulnerability, to better understand potential predisposing factors.

Malignant catatonia requires prompt therapeutic action. Lorazepam, a recognised first-line benzodiazepine,^26^ was administered. An insufficient initial response prompted dose escalation and ECT, resulting in notable improvements in mobility and communication over 31 sessions, consistent with ECT’s high efficacy.^27^ The subsequent deterioration following ECT discontinuation illustrates catatonia’s potential chronicity and relapsing nature, reflecting neurobiological persistence as documented in longitudinal studies of malignant presentations.^25^ A collaborative multidisciplinary approach involving psychiatric, neurological and rehabilitative teams was essential for addressing this complex situation. This integrated method, particularly through the application of ECT, played a crucial role in the patient’s recovery. The lingering symptoms and continuous need for specialised follow-up care underscore a key principle in managing catatonia: positive outcomes rely on sustained, comprehensive treatment strategies.^30^

This case has limitations, such as the ambiguous role of the POGZ gene variant to the clinical phenotype and the challenge of reaching a definitive diagnosis due to overlapping psychiatric and neurological symptoms. Future studies should explore how POGZ mutations affect neurodevelopment and psychiatric susceptibility, including their relationship with specific neuropsychiatric presentations and the long-term effectiveness of treatments like ECT for patients with genetic predispositions.

This report of malignant catatonia in an adolescent with a POGZ gene mutation highlights key aspects of treatment-resistant neuropsychiatric disorders. The patient’s journey emphasises the need for early detection and coordinated care. ECT offered vital but temporary relief, underscoring the need for sustained long-term treatment. Identifying the POGZ variant contributes to understanding potential genetic influences on neuropsychiatric vulnerability and their relevance in diagnostically complex cases. While not currently enabling POGZ-specific personalised catatonia treatment, this finding underscores the need for further research into the genetic architecture of such disorders to potentially develop targeted interventions. The CARE Checklist for this case report is in the online Supplementary material available at https://doi.org/10.1192/bjo.2025.10807.

## Supporting information

Leibovitch et al. supplementary material Leibovitch et al. supplementary material

## Data Availability

All the data generated or analysed during this study are included in this article. The data supporting this study’s findings are not publicly available because they contain information that could compromise the privacy of the research participants. However, the data are available from the corresponding author upon reasonable request.

## Supplementary material

The supplementary material is available online at https://doi.org/10.1192/bjo.2025.10807
