# Supplementary material for: Malignant catatonia in an adolescent with pogo transposable element derived with zinc finger domain (POGZ) gene mutation: case report
Source: BJPsych Open. 2025 Aug 1;11(5):e170. doi: 10.1192/bjo.2025.10807 (PMC12344435; doi:10.1192/bjo.2025.10807)
Supplement: Leibovitch et al. supplementary material [file S2056472425108077sup001.docx]

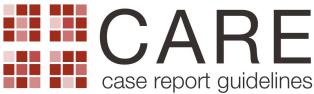
**CARE Checklist (2013) of information to include when writing a case report
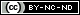
**

**Topic Item Checklist item description Reported on Page**

**Title 1** The words “case report” should be in the title along with the area of focus Page 1

**Key Words 2** 2 to 5 key words that identify areas covered in this case report Page 1

**Abstract 3a** Introduction—What is unique about this case? What does it add to the medical literature? Page 2

**3b** The main symptoms of the patient and the important clinical findings Page 2

**3c** The main diagnoses, therapeutics interventions, and outcomes Page 2

**3d** Conclusion—What are the main “take-away” lessons from this case? Page 2

**Introduction 4** One or two paragraphs summarizing why this case is unique with references Page 3

**Patient Information 5a** Demographic information and other patient specific information Page 4

**5b** Main concerns and symptoms of the patient Page 4-6

**5c** Medical, family, and psychosocial history including relevant genetic information (also see timeline). Page 4

**5d** Relevant past interventions and their outcomes Page 4

**Clinical Findings 6** Describe the relevant physical examination (PE) and other significant clinical findings Page 4-6

**Timeline 7** Important information from the patient’s history organized as a timeline Page 4-6; Table 1

# Diagnostic Assessment

**Therapeutic Intervention**

**Follow-up and Outcomes**

**8a** Diagnostic methods (such as PE, laboratory testing, imaging, surveys) Page 4,5

**8b** Diagnostic challenges (such as access, financial, or cultural) N/A

**8c** Diagnostic reasoning including other diagnoses considered Page 4-5,7

**8d** Prognostic characteristics (such as staging in oncology) where applicable N/A

**9a** Types of intervention (such as pharmacologic, surgical, preventive, self-care) Page 4-6

**9b** Administration of intervention (such as dosage, strength, duration) Page 4-6

**9c** Changes in intervention (with rationale) Page 4-5

**10a** Clinician and patient-assessed outcomes (when appropriate) Page 6

**10b** Important follow-up diagnostic and other test results Page 5-6

**10c** Intervention adherence and tolerability (How was this assessed?) N/A

**10d** Adverse and unanticipated events Page 4-5

**Discussion 11a** Discussion of the strengths and limitations in your approach to this case Page 8

**11b** Discussion of the relevant medical literature Page 3,7

**11c** The rationale for conclusions (including assessment of possible causes) Page 8

**11d** The primary “take-away” lessons of this case report Page 8

**Patient Perspective 12** When appropriate the patient should share their perspective on the treatments they received N/A

**Informed Consent 13** Did the patient give informed consent? Please provide if requested . . . . . . . . . . . . . . . . . . . . . . . . . . . . . . . . . . . .. . YES
